# Digital advances for multicentre studies – the British Urology Researchers in Surgical Training (BURST) experience

**DOI:** 10.1111/bju.70447

**Published:** 2026-09-17

**Authors:** Nikki Kerdegari, Feargus Hosking‐Jervis, Veeru Kasivisvanathan, Kevin Byrnes, Kevin Gallagher, Sinan Khadhouri, Nikita Bhatt

**Affiliations:** ^1^ Guy's and St Thomas’ NHS Foundation Trust London UK; ^2^ British Urology Researchers in Surgical Training (BURST) Research Collaborative London UK; ^3^ GKT School of Medical Education King's College London London UK; ^4^ Division of Surgery and Interventional Science University College London London UK; ^5^ Department of Urology Western General Hospital, University of Edinburgh Edinburgh UK; ^6^ Department of Urology University of Glasgow and Glasgow Royal Infirmary Glasgow UK; ^7^ Department of Urology St Vincent's University Hospital Dublin Ireland

**Abbreviations**
app: application
BURST: British Urology Researchers in Surgical Training
REDCap: Research Electronic Data Capture
WASHOUT: Ward AdmiSsion of Haematuria: an Observational mUlticentre sTudy

The UK National Institute for Health and Care Research (NIHR) spent £578.21 million funding research programmes to improve patient outcomes in 2024/2025 [1]. Despite considerable investment, >40% of UK trials fail to achieve their intended recruitment [2]. Collaborative multicentre research is valuable in the evolving research landscape. It can answer questions that traditional trials cannot. A large network of international collaborating sites allows a shorter time to sufficient power, whilst maintaining methodological robustness. The collaborative model increases accessibility in participation, overcoming barriers such as financial constraints, geography, and lack of infrastructure. The increased sample sizes, data granularity and diversity of participants create the potential to give high certainty answers to research questions whilst meeting the needs of increasingly diverse and complex patient populations.

A barrier to multicentre studies is the logistical challenge of coordinating many parties. With over a decade of experience, and in partnership with database developers, the British Urology Researchers in Surgical Training (BURST) collaborative has evolved to adopt innovations to improve site participation and streamline data entry whilst maintaining high data quality. This has resulted in large‐scale, meaningful outputs. In the IDENTIFY study (ClinicalTrials.gov identifier: NCT03548688), 10 896 patients from 110 hospitals were recruited in 12 months [3]. In the RESECT study (NCT05154084), an embedded cluster randomised controlled trial was enabled, randomising 220 sites in 18 months and collecting data about 19 505 transurethral resection of bladder tumour cases from 230 sites across all phases of the study in 27 months [4].

Research Electronic Data Capture (REDCap) is a locally hosted, non‐commercial data management platform accessible from a web browser and widely used internationally. BURST uses REDCap for site registration, data entry, and data management for the study team. The experience for participating sites is improved by simplifying the registration process and self‐service of input, such as surgeon demographic data.

Applications (apps) can be connected to REDCap on top of its core database structure. We have made use of this functionality to build study‐specific apps, developed using ‘Shiny’ (an R web app framework), which are distributed to relevant users. Apps include: The RESECT surgical performance feedback dashboard, an authorship and participant certificate management app and a data quality app (Figs S1–S3). The data quality app was developed during RESECT and refined for our most recent study the ‘Ward AdmiSsion of Haematuria: an Observational mUlticentre sTudy’ (WASHOUT). The app automatically extracts data in real‐time and on‐demand, processes and standardises it, and presents site‐specific data quality reports to users. This automated data quality check is integrated with ratification, making it simple for sites to rectify errors promptly and overcome inconsistencies in data reporting. The authorship app calculates authorship requirements for collaborative work, automates authorship generation for manuscripts, and generates individual certificates. The use of automation in study administration saves time for BURST and participating sites.

Leveraging on friendly competitiveness, studies have an accompanying leaderboard ranking sites based on key performance indicators such as the number of cases entered into the REDCap database (Fig. 1) and the number of cases with complete follow‐up. The leaderboard is automatically updated and publicly accessible through the BURST website (can be integrated into any website). This allows top‐performing sites to be celebrated and acts as a motivational tool for collaborators with benchmarking and incentivises continued engagement in providing high‐quality data and adherence to the methodology. This ensures the case report form is compliant with the study protocol.

**Fig. 1:**
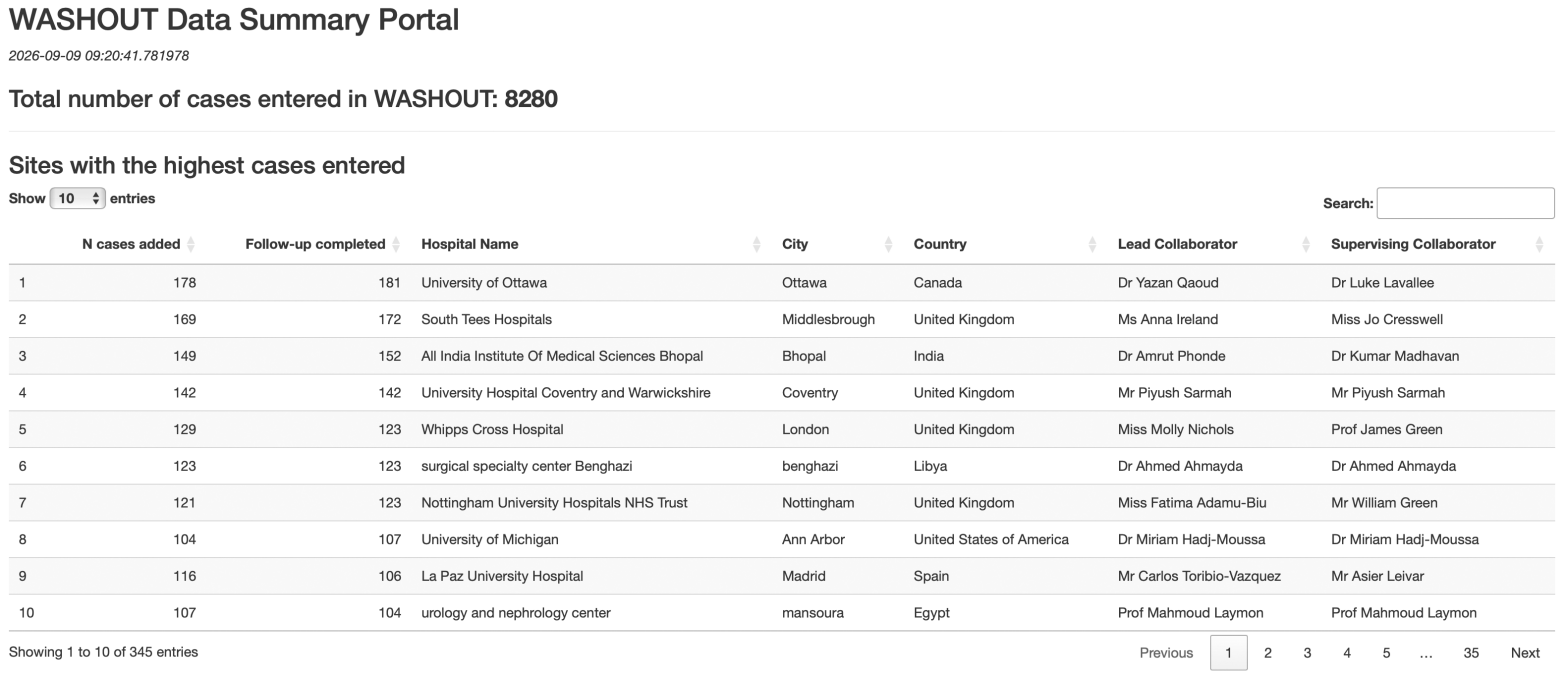
The WASHOUT leaderboard of recruiting sites displaying the top recruiting sites according to the number of cases added to the study.

Maintaining a strong working relationship with collaborators is fundamental. A strong social media presence helps increase the number of recruited sites and enables real‐time communication of updates and deadlines. Sites are also sent fortnightly automated bespoke e‐mails, including personalised content. This includes global ranking, continental ranking and national ranking for the key metrics of cases entered, cases with completed follow‐up, and data quality completeness. We have a total of five e‐mail templates that are used for site e‐mails, depending on the difficulty of the task (Figs S1–S3). This creates a more personalised experience for collaborators and provides meaningful comparison with nearby centres, providing achievable goals that maintain their vested interest in the study.

Furthermore, in‐house digital advancements create less dependence on expensive, proprietary software and database providers, a trend that is becoming even more pronounced in the artificial intelligence era. The use of remote, routine health record data collection minimises barriers to participation and fosters enhanced representation of typically underrepresented demographics within studies, increasing potential generalisability of results across diverse populations [5], whilst significantly reducing cost compared to traditional clinical trial ‘case report form’‐based data collection. The application of such advancements must always occur alongside adherence to the latest privacy and data protection regulations along with secure data transmission [6]. The BURST collaborative model has successfully demonstrated that it is possible to collect large‐scale meaningful datasets quickly whilst complying with regulations.

Potential barriers when adopting digital technologies in multicentre research must also be considered before implementation, e.g., fragmented communication [7]. Whilst this has not been a challenge in our own institutional experience, there is a potential role for training local clinical staff as ‘technology champions’ to improve dissemination and use of digital tools and usability testing for the refinement of these tools [8]. This could translate to a REDCap champion at each participating BURST site.

In conclusion, the integration of digital advancements into the BURST collaborative research model have helped overcome the traditional challenges of collaborative research, including administrative burden, data quality assurance, site engagement, and cost. The combination of self‐service data entry, automated data quality reporting, and performance benchmarking is a contributory factor to the success seen in the scale of BURST's collaborative research. Other researchers may be interested in adoption of such digital approaches to large‐scale multicentre research to enhance delivery of high‐quality and efficient studies.

## Disclosure of Interests

The author Feargus Hosking‐Jervis was employed by the company Urologistics Ltd. The remaining authors confirm that this research was undertaken without any commercial or financial relationships that might constitute a potential conflict of interest.

## Supporting information


**Fig. S1.** Data quality app displaying summary error counts and a record‐level breakdown of detected data errors, each linked directly to the corresponding entry in REDCap for correction.
**Fig. S2.** Authorship app listing verified collaborators (with name, affiliation, Open Researcher and Contributor identification [ORCID] code, and designation) and the site's inclusion status for the primary paper. The authorship app calculates authorship requirements, automates authorship generation for manuscripts and generates individual certificates.
**Fig. S3.** Example automated e‐mail sent to sites that have completed authorship verification, showing their verified author count, progress bar, and global/regional ranking.

## Data Availability

Data sharing not applicable to this article as no datasets were generated or analysed during the present study.
