# Supplementary material for: Digital advances for multicentre studies – the British Urology Researchers in Surgical Training (BURST) experience
Source: BJU Int. 2026 Sep 17;138(5):747–9. doi: 10.1111/bju.70447 (PMC13655206; doi:10.1111/bju.70447)
Supplement: Supplementary file 1 [file BJU-138-747-s001.docx]

**Supplementary Material**

*Figure 1: Data quality app displaying summary error counts and a record-level breakdown of detected data errors, each linked directly to the corresponding entry in REDCap for correction*

*Figure 2: Authorship App listing verified collaborators (with name, affiliation, ORCID, and designation) and the site's inclusion status for the primary paper. The authorship app calculates authorship requirements, automates authorship generation for manuscripts and generates individual certificates.*

*Figure 3: Example automated email sent to sites that have completed authorship verification, showing their verified author count, progress bar, and global/regional ranking.*
